# NUP37 promotes the proliferation and invasion of glioma cells through DNMT1-mediated methylation

**DOI:** 10.1038/s41420-024-02138-5

**Published:** 2024-08-22

**Authors:** Yongqiang Lv, Chaolian Wang, Ruoyu Liu, Shaoxian Wu, Junjun Chen, Xiao Zheng, Tianwei Jiang, Lujun Chen

**Affiliations:** 1 Department of Neurosurgery, The Third Affiliated Hospital of Suzhou University, Changzhou, Jiangsu China; 2 Department of Tumor Biological Treatment, The Third Affiliated Hospital of Suzhou University, Changzhou, Jiangsu China; 3 Jiangsu Engineering Research Center for Tumor Immunotherapy, The Third Affiliated Hospital of Suzhou University, Changzhou, Jiangsu China; 4 Institute of Cell Therapy, The Third Affiliated Hospital of Suzhou University, Changzhou, Jiangsu China

**Keywords:** CNS cancer, Methylation

## Abstract

Nuclear regulation has potential in cancer therapy, with the nuclear pore complex (NPC) serving as a critical channel between the nucleus and cytoplasm, playing a role in regulating various biological processes and cancer. DNA methylation, an epigenetic modification mediated by DNA methyltransferases (DNMTs), influences gene expression and cell differentiation, and is crucial for the development and progression of tumor cells. Gliomas are the most common primary brain tumors, with glioblastoma being particularly aggressive, characterized by invasiveness, migration capability, and resistance to conventional treatments, resulting in poor prognosis. Our study revealed that the expression level of NUP37 affects the proliferation and invasion of glioma cells, and that the overexpression of DNMT1 can alleviate the adverse effects caused by NUP37 depletion. These findings suggest that NUP37 promotes the proliferation and invasion of glioma cells through its interaction with DNMT1.

## Introduction

Glioma represents the most common type of primary brain tumor, accounting for 28% of all such tumors. Alarmingly, they constitute 80% of all malignant primary brain tumors in adults and are typically associated with a bleak prognosis [1]. Specifically, glioblastoma, with its high malignancy, is marked by invasiveness, migration abilities, and resistance to standard treatments, often leading to a short survival period post-diagnosis [2, 3]. Conventional treatment strategies for glioma mainly include surgical resection, radiotherapy, and chemotherapy. However, these treatments frequently induce various neurological symptoms and additional discomfort, severely impacting patients’ quality of life [4]. The recent emergence of tumor biological immunotherapy and molecular targeting treatments, such as PD-1/PD-L1 immunological checkpoints, monoclonal antibodies, and chimeric antigen receptor T cell immunotherapy, has instilled renewed hope for cancer patients, including those suffering from glioma. There are encouraging signs that such tumor therapies harbor the significant potential for glioma treatment. Consequently, there’s an urgent necessity to identify and explore novel tumor biomarkers, thus broadening the possibilities for glioma immunotherapy and targeted treatments.

Nuclear regulation has increasingly been recognized as a promising target for cancer treatment due to its critical role in cellular processes. The nuclear envelope, acting as a boundary separating the nucleus from the cytoplasm, is responsible for managing mRNA and ribosomal protein transportation [5, 6]. Comprising over 30 nucleoporins, the nuclear pore complex (NPC) oversees the movement of molecules across the nuclear envelope. NPCs participate in regulating diverse biological processes (BP), including cell cycle progression, apoptosis, and cell migration [7]. Of particular note among the multitude of NPCs, the Nup107 sub-complex plays a crucial role as it impacts gene expression, heterochromatin function, and nuclear envelope formation [8]. Nup37, a constituent of the Nup107 sub-complex in yeast [9, 10], has been observed to possess tumor-suppressive properties in oral squamous cell carcinoma [11]. Research conducted by Luo et al. has revealed that NUP37 overexpression fosters the growth of hepatocellular carcinoma (HCC), whereas suppressing NUP37 expression curtails the proliferation and metastasis of HCC both in vivo and in vitro [12]. Similarly, studies by Huang et al. have suggested that overexpression of NUP37 in non-small cell lung cancer (NSCLC) plays a part in regulating apoptosis and cell cycle control [13]. Furthermore, Haskell et al. have unearthed a potential link between NUP37 gene mutations and cardiovascular diseases. These studies hint at a possible regulatory function of NUP37 in human cancer [14]. However, the precise roles and significance of NUP37 in glioma are yet to be comprehensively explored.

DNA methylation, enabled by DNA methyltransferases (DNMTs), is a fundamental epigenetic modification that plays a pivotal role in regulating gene expression and controlling cellular differentiation [15]. In mammals, DNMTs preserve DNA methylation patterns by attaching methyl groups to the 5-carbon positions of cytosines in CpG dinucleotides. There’s a growing body of evidence suggesting that abnormal DNA hypermethylation in promoter regions leads to the silencing of tumor suppressor genes [16, 17]. DNMT1, a key methyltransferase encoded by the *DNMT1* gene situated on chromosome 19p13.2, is tasked with maintaining DNA methylation patterns. DNMT1 overexpression has been observed in a variety of malignant tumors, including gliomas [18, 19]. However, the precise relationship between NUP37 and DNMTs, as well as the mechanism that underpins their interaction in impacting glioma expression, remains largely elusive.

In the present study, we conducted an in-depth examination of the expression levels of NUP37 and DNMT1 in gliomas. Through in vitro and in vivo cellular functional experiments, we established that NUP37 depletion effectively hindered the proliferation, invasion, and other cellular activities of glioma cells. Subsequent bioinformatics analysis and co-immunoprecipitation (Co-IP) experiments furnished robust evidence of the interaction and binding between NUP37 and DNMT1. Notably, DNMT1 overexpression successfully mitigated the impaired proliferation, invasion, and migration of glioma cells instigated by NUP37 depletion. Collectively, these findings revealed the role of NUP37 in fostering the proliferation, invasion, and migration of glioma cells, primarily via its interaction with DNMT1.

## Results

### NUP37 is enriched in gliomas and regulates biological functions

**Fig. 1:**
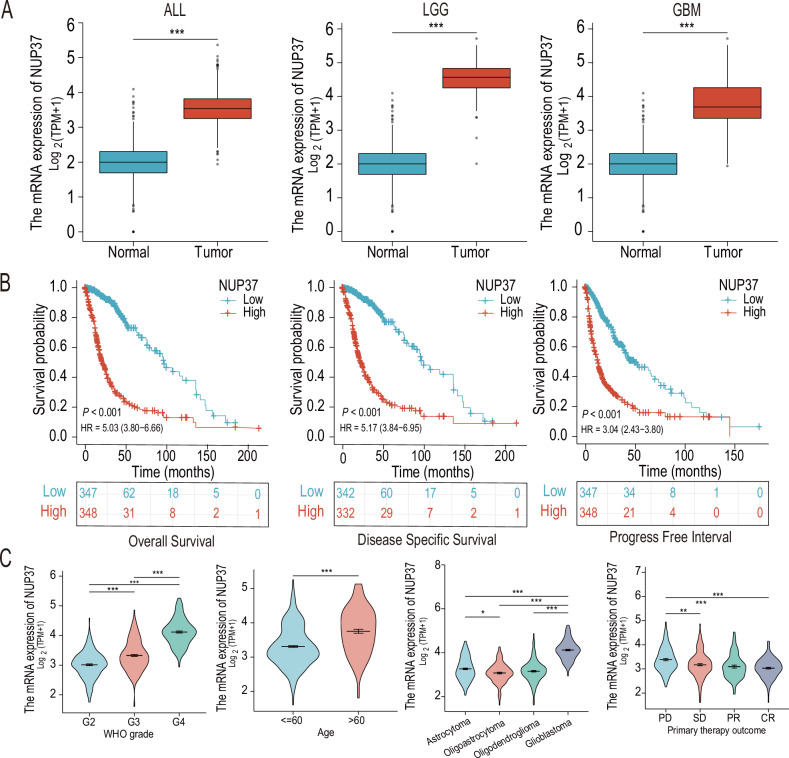
NUP37 is overexpressed in gliomas and is closely associated with a number of clinical features. **A** The expression level of NUP37 in glioma was analyzed based on TCGA and GTEx public databases. **B** Kaplan–Meier survival analysis was used to evaluate the prognostic role of NUP37 in gliomas. **C** The correlation between NUP37 expression level and clinical characteristics of glioma was analyzed. The significance level was set as follows: *P < 0.05, **P < 0.01, and ***P < 0.001.

By using The Cancer Genome Atlas (TCGA) database, we observed that the expression of NUP37 was significantly higher in both low-grade and high-grade gliomas compared to normal brain tissues (Fig. 1A). Kaplan–Meier survival analysis indicated that glioma patients with high NUP37 expression experienced significantly lower overall survival, disease-specific survival, and progression-free survival than those with low NUP37 expression (Fig. 1B). Moreover, clinical correlation analysis revealed a positive correlation between NUP37 expression levels and the World Health Organization (WHO) grade of glioma. Specifically, the G4 glioma exhibited the highest NUP37 expression, while the G2 glioma showed the lowest expression. Additionally, the expression level of NUP37 was found to correlate with clinical characteristics, such as patient age, histopathological type, and treatment outcomes (Fig. 1C).

**Fig. 2:**
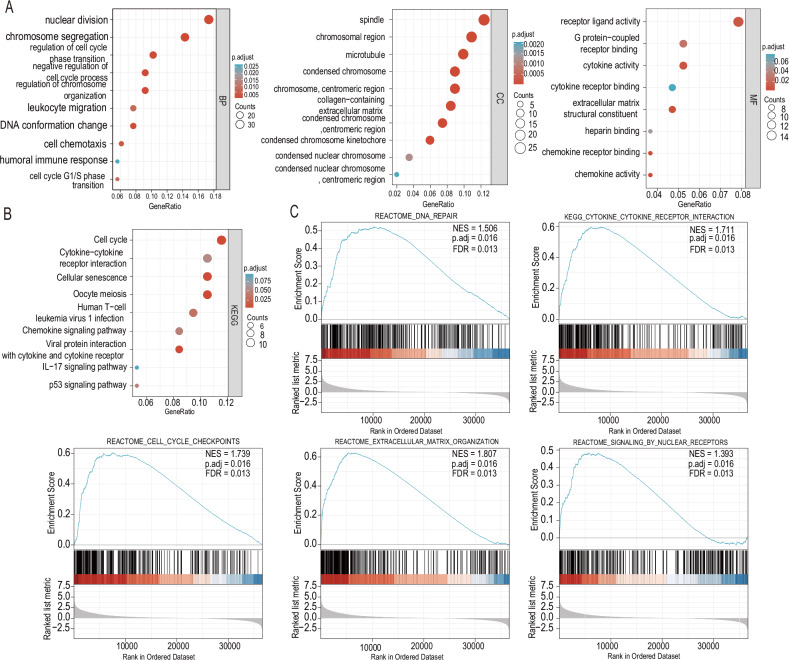
Enrichment analysis of NUP37 was closely associated with glioma cell function and signaling pathways. **A**–**C** GO, KEGG, and GSEA enrichment analyses were performed for NUP37 co-expressed genes, respectively.

To elucidate the expression relationship between NUP37 and other genes, we conducted a bioinformatics analysis of NUP37 in glioma using its co-expressed genes. The top 300 genes associated with NUP37 were extracted from the TCGA database, and several analyses were performed using R software. Gene Ontology (GO) analysis revealed that NUP37 was implicated in various BP, including the regulation of neutrophil degranulation, glycoprotein metabolic process, negative regulation of N-linked glycosylation of cell cycle process proteins, late promoting complex-dependent catabolic process, transferring glycosyl, and transferring hexosyl. Moreover, it was discovered that NUP37 was associated with DNA replication in the cell cycle process (Fig. 2A). Kyoto Encyclopedia of Genes and Genomes (KEGG) pathway analysis identified several pathways involving NUP37, such as N-sugar biosynthesis, DNA replication, ER protein processing, biosynthesis of various N-sugars, mismatch repair, and nucleotide excision repair (Fig. 2B). To delve deeper into the function of NUP37 in glioma, we carried out Gene Set Enrichment Analysis (GSEA) using TCGA data. The analysis highlighted the enrichment of pathways, such as cytokine receptor interactions and cell cycle checkpoints, in the KEGG pathway, DNA repair, extracellular matrix organization in the Reactome pathway, and ECM regulation in the NABA matrix (Fig. 2C).

### NUP37 is overexpressed in glioma tissue samples

**Fig. 3:**
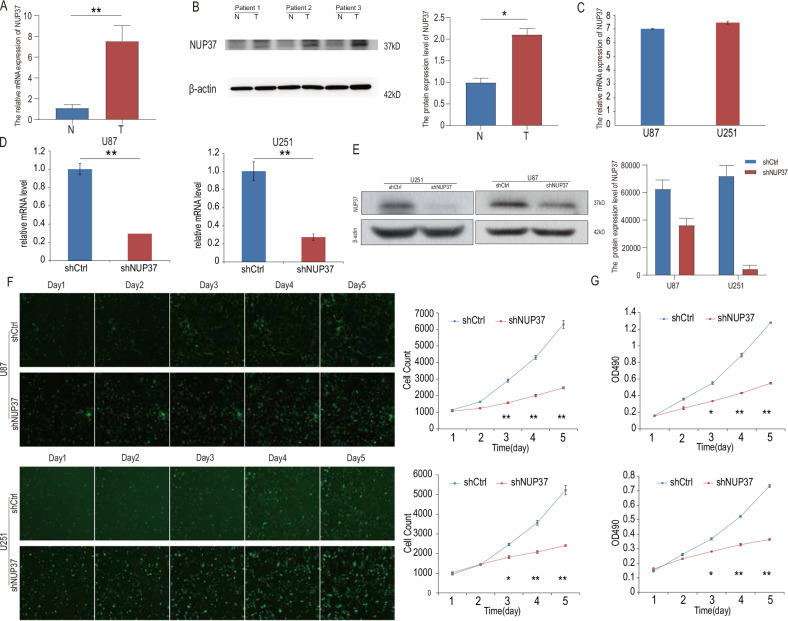
NUP37 expression and construction of NUP37 depletion cell lines. **A**, **B** qPCR and western blotting analysis were used to verify the expression of NUP37 in glioma tissue samples. **C** The expression levels of endogenous NUP37 in primary U87 and U251 glioma cells were detected. **D**, **E** qPCR and western blotting analysis were used to verify the efficiency of NUP37 depletion. **F**, **G** MTT proliferation assay and cell counting assay were used to observe the proliferation activity of U87 and U251 glioma cells after NUP37 depletion. The significance level was set as follows: *P < 0.05, **P < 0.01.

The WHO classifies gliomas into several grades. Grades II and III were defined as low-grade glioma. To investigate the expression of NUP37 at both the mRNA and protein levels, we obtained tumor tissue from 35 patients with gliomas and normal brain tissue from 15 patients who had experienced traumatic brain injury. These tissues have been stored in the laboratory for an extended period of time. Total RNA and total protein were extracted from the tissues using Trizol and RIPA reagents, respectively. Subsequently, we conducted qPCR experiments to measure the expression of NUP37 at the mRNA level. Additionally, western blotting analysis was performed to analyze the expression of NUP37 at the protein level. The results demonstrated that NUP37 was expressed more prominently in glioma tissues compared to normal brain tissues (Fig. 3A, B).

### Depletion of NUP37 exerts different effects on multiple biological co-functions of glioma cells

The endogenous expression of NUP37 in glioma cell lines was determined using a qPCR assay. To investigate the role of NUP37 in brain glioma cell biology, glioma cells were transfected with lentivirus to construct a stable cell line with low expression of NUP37. Subsequently, qPCR and western blotting analysis were utilized to confirm the efficiency of the depletion (Fig. 3C–E). The MTT assay was conducted to explore the potential effects of NUP37 depletion on cell proliferation in U87 and U251 cells. We observed that cell growth was significantly inhibited in cells transfected with shNUP37 compared to those transfected with shCtrl (*P* < 0.05) (Fig. 3G). This finding suggested a significant reduction in the proliferation rate upon NUP37 depletion in both U87 and U251 cells.

Furthermore, the Celigo cell counting method was employed to directly monitor and quantify the proliferation of U87 and U251 cells. After infecting both cell lines with shNUP37 or shCtrl, EGFP-positive signals were identified. After 5 days of culture, the green signals in U87 and U251 cells infected with shNUP37 were markedly lower compared to those infected with shCtrl (Fig. 3F).

**Fig. 4:**
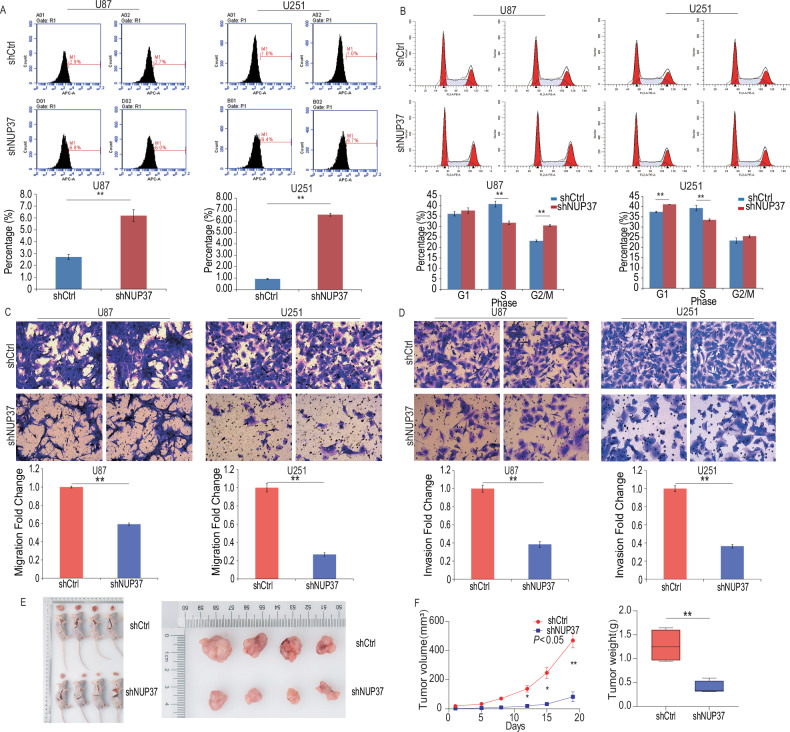
Depletion of NUP37 inhibits the biological function and tumorigenicity of glioma cells in vivo. **A** The apoptosis rate of U87 and U251 glioma cells was detected by flow cytometry. **B** Flow cytometry was used to detect the proportion of U87 and U251 glioma cells in each cycle. **C**, **D** Transwell invasion and migration assay was used to detect the invasion and migration ability of U87 and U251 glioma cells. **E**, **F** To establish an ectopic tumor model in mice to observe the tumorigenic effect of glioma cells. The significance level was set as follows: **P < 0.01.

To determine the percentage of apoptosis in U87 and U251 cells transfected with shNUP37, flow cytometry analysis was performed. The results revealed an increase in apoptosis induction in U87 and U251 cells transfected with shNUP37. The average percentage of apoptotic U87 cells was 6.23%, significantly higher than that in the shCtrl group (2.7%). Similarly, the average percentage of apoptotic U251 cells was 6.57%, significantly higher than that in the shCtrl group (0.97%) (Fig. 4A).

Flow cytometry analysis demonstrated that the depletion of NUP37 led to notable alterations in the cell cycle of U87 and U251 cells. In U87 cells, NUP37 depletion resulted in a significant reduction of cells in the S phase, accompanied by an accumulation in the G2/M phase. In contrast, in U251 cells, NUP37 depletion prompted a decrease in the S phase and an accumulation of cells in the G1 phase.

Specifically, in U87 cells treated with shNUP37, the percentage of cells in the S phase was 31.81%, significantly lower than the shCtrl group (40.77%). Additionally, there was an accumulation of cells in the G2/M phase, with about 31.44% of cells blocked at this phase, compared to the control cells (22.93%). In U251 cells, the proportion of cells in the G2/M phase was 23.63% in the control group, whereas it was increased to 26.13% in the group treated with shNUP37. The percentage of cells in the S phase was 32.94% in shNUP37-treated U251 cells, significantly lower than the shCtrl group (38.87%). Furthermore, an accumulation of cells was observed in the G1 phase, with ~41.06% of cells blocked at this phase after shNUP37 treatment, compared to the control cells (37.03%) (Fig. 4B).

In the Transwell invasion assay, we observed that the number of migrating cells in the shNUP37 group was significantly lower compared with the shCtrl group, indicating a significant difference. This finding suggested that the depletion of NUP37 expression resulted in a decrease in the invasive ability of U87 and U251 cells (Fig. 4C). Moreover, the depletion of NUP37 in glioma cell lines led to a significant reduction in their metastatic capability. In the Transwell metastasis experiment, the number of migrating cells in the shNUP37 group was significantly lower compared to the shCtrl group, presenting a statistically significant difference (Fig. 4D). These results implied that the metastatic and invasive abilities of U87 and U251 cells were diminished following the depletion of NUP37.

To further explore the function of NUP37 in glioma in vivo, subcutaneous tumor formation experiments were carried out using mouse models bearing tumors. Glioma cells transfected with lentivirus were subcutaneously implanted into 4-week-old nude mice, and the progression of tumor growth was regularly monitored and measured. The results revealed that the subcutaneous tumor volume in the experimental group (shNUP37), in which the NUP37 gene was stable low expression, was smaller than that in the control group (shCtrl) on the 12th, 15th, and 19th days of measurement, respectively. Additionally, after the mice were euthanized, the weight of the subcutaneous tumors was recorded and analyzed. The results demonstrated that the weight of the subcutaneous tumor in the experimental group (shNUP37) was significantly lower than that in the control group (shCtrl) (Fig. 4E). This difference in tumor volume and weight between the experimental and control groups was statistically significant (*P* < 0.05). These findings highlighted the crucial role that NUP37 might play in glioma development and progression in vivo.

### Results of transcriptional sequencing combined with proteomic sequencing

**Fig. 5:**
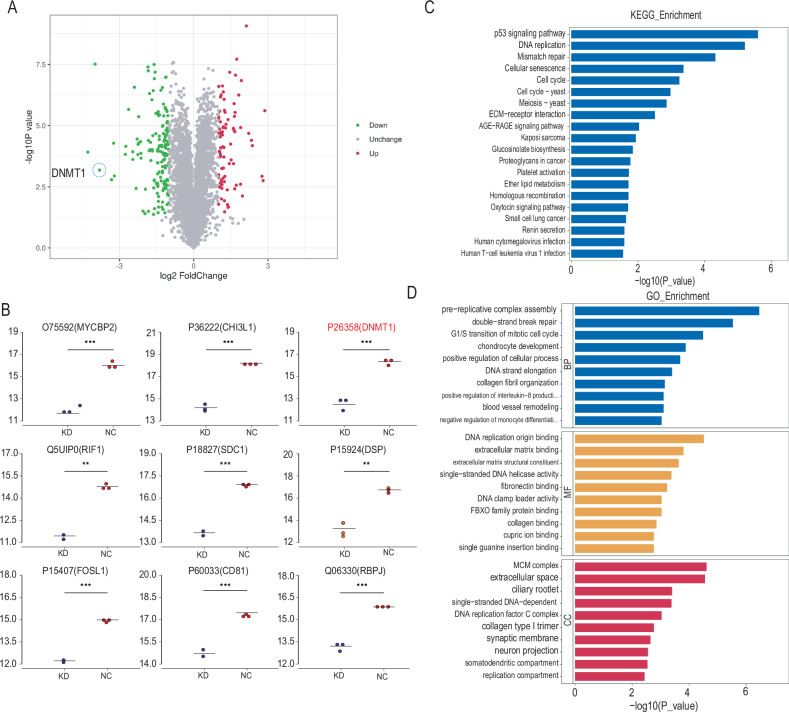
After the depletion of NUP37, the expression of some genes in glioma cells changes to different degrees. **A** Volcano plot of DEGs in glioma cells after NUP37 depletion. **B**–**D** KEGG pathway enrichment analysis, Reactome enrichment analysis, and GO functional enrichment analysis were performed on DEGs. The significance level was set as follows: **P < 0.01, and ***P < 0.001.

The results of differential gene expression analysis following the depletion of NUP37 in glioma cells are illustrated in Fig. 5A. A total of 1350 genes exhibited significant changes in their expression levels, out of which 989 genes were downregulated, and 361 genes were upregulated. Following this, KEGG pathway enrichment analysis and GO functional enrichment analysis were performed on these differentially expressed genes (DEGs). KEGG analysis revealed enrichment in several pathways, including cell cycle, DNA replication, cell aging, mismatch repair, meiotic homologous recombination, and p53 signaling pathway. Furthermore, Reactome enrichment analysis was carried out for these DEGs (Fig. 5B). GO functional enrichment analysis revealed several affected BP, cellular components (CC), and molecular functions (MF) (Fig. 5C). Under BP, processes like chromosome segregation, sister chromatid separation, nuclear chromosome separation, DNA replication, and nuclear division were affected. For CC, changes were observed in chromosomes, centromere regions, and the spindle. As for MF, there was altered catalytic activity on DNA, chromatin binding, and microtubule binding.

Upon NUP37 depletion in U87 glioma cells, significant decreases were observed in various biological pathways, including cell cycle checkpoints, DNA strand elongation in DNA replication, activation of pre-replication complexes, G1–G1/S phase progression in mitosis, G1–S phase transition, S phase of mitosis, DNA synthesis, and centrosome signal amplification. These changes suggested a regulatory role for NUP37 in these processes. Moreover, apart from the aforementioned decreased biological pathways, an increase was noted in some signaling pathways following the NUP37 depletion. These included the RNA polymerase 1 promoter pathway, DNA methylation pathway, SIRT1 negative regulation of rRNA expression pathway, and DNA damage/telomere stress-induced aging pathway. These findings suggested that NUP37 might have regulatory effects on these signaling pathways in U87 glioma cells.

After NUP37 depletion in glioma cells, disease-related enrichment analysis using the Disease Ontology and DisGeNET databases was performed on the DEGs. Our data revealed a significant reduction in the expression of various cancer-associated signaling pathways, including nervous system cancer, ocular cancer, endocrine gland tumors, ovarian epithelial origin cancer, ovarian cancer, retinal cell carcinoma, retinoblastoma, autonomic nervous system tumor, breast cancer, pancreatic cancer, and cancers in other organ systems. Additionally, a notable decrease was observed in the expression of multi-system cancer-related pathways in specific types of cancer, such as triple-negative breast cancer, chromosome breakage, progranulocytosis, hematological tumors, retinoblastoma, Fanconi anemia, and adenoid cystic carcinoma. These results suggested a potential role for NUP37 in regulating these cancer-related pathways in glioma cells.

**Fig. 6:**
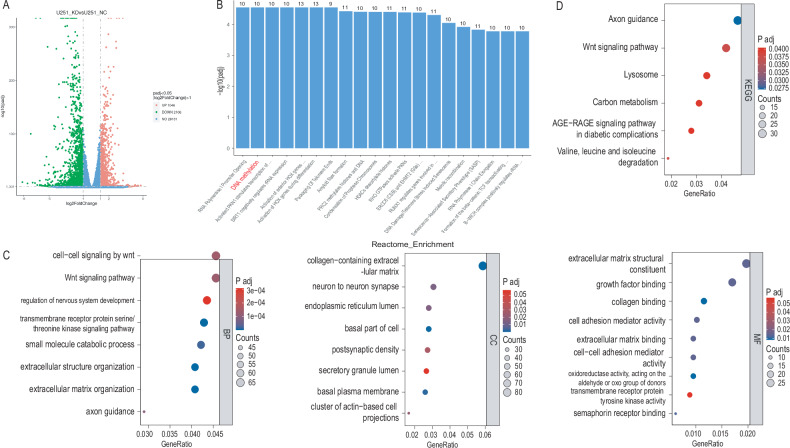
After the depletion of NUP37, the expression of protein levels and multiple biological signaling pathways in glioma cells changes to different degrees. **A** Volcano plot of differentially expressed proteins in glioma cells after NUP37 depletion. **B** The top nine significantly downregulated proteins were screened out. **C**, **D** GO and KEGG enrichment analyses were performed on differentially expressed proteins.

Upon NUP37 depletion, protein expression analysis revealed significant changes in a total of 673 proteins, with 238 upregulated proteins and 435 downregulated proteins (Fig. 6A). Among the differentially expressed proteins, the six most significantly altered were selected for further analysis. The downregulated proteins identified included MYCBP2, CHI3L1, DNMT1, RIF1, SDC1, and DSP (Fig. 6B), which all exhibited reduced expression levels following NUP37 depletion. Moreover, GO and KEGG enrichment analyses were performed for the significantly differentially expressed proteins. The results of the GO enrichment analysis included BP, such as the assembly of replication precursors, G1/S phase transition of cell mitosis, and positive regulation of cellular processes. For CC, alterations were noted in the MCM complex, DNA replication factor complex C, extracellular space, cell ciliary root filament, and type I collagen trimer. In terms of MF, changes were observed in DNA replication origin binding, extracellular matrix binding, extracellular matrix structural components, fibronectin binding, and single-stranded DNA helicase activation. KEGG enrichment analysis revealed changes in pathways like DNA replication, cell aging, cell cycle, extracellular matrix–receptor interaction, proteoglycans in cancer, and the p53 signaling pathway (Fig. 6C, D). These GO and KEGG enrichment results were largely consistent with the transcriptome sequencing bioinformatics analysis presented in the first part of this study.

### DNMT1 is highly expressed in gliomas and regulates biological functions

**Fig. 7:**
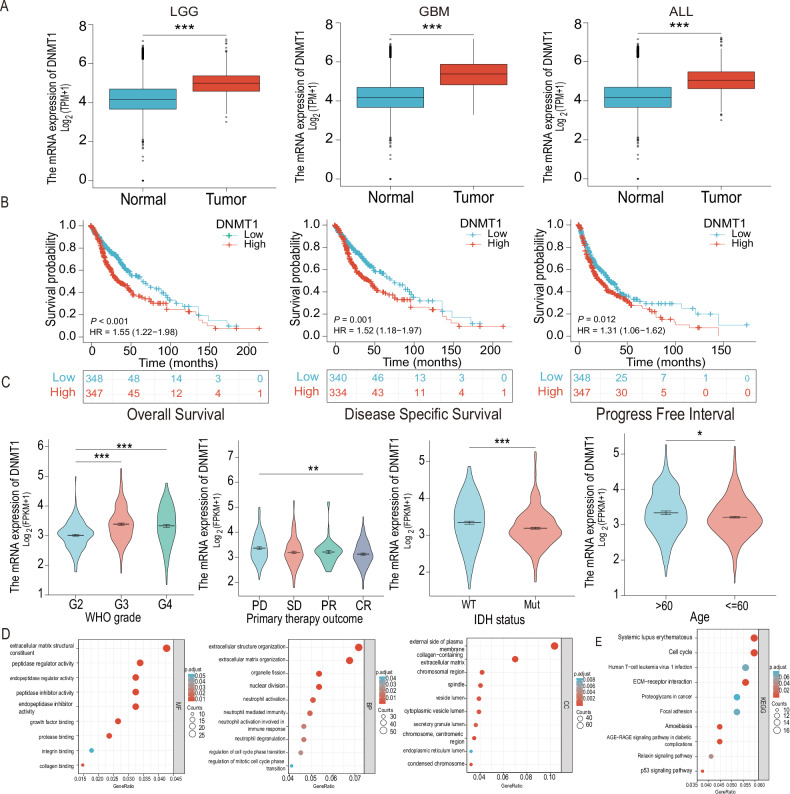
NUP37 is overexpressed in gliomas and is closely associated with a number of clinical features, cell functions, and signaling pathways in gliomas. **A** The expression level of DNMT1 in glioma was analyzed based on TCGA and GTEx public databases. **B** Kaplan–Meier survival analysis was used to evaluate the prognostic role of DNMT1 in gliomas. **C** The correlation between DNMT1 expression level and clinical characteristics of glioma was analyzed. **D**, **E** GO and KEGG enrichment analyses were performed on DNMT1 co-expressed genes. The significance level was set as follows: *P < 0.05, **P < 0.01, and ***P < 0.001.

TCGA database was utilized to examine the differential expression of DNMT1 in gliomas. We found an elevated expression of DNMT1 in both low-grade and high-grade gliomas when compared to normal brain tissue (Fig. 7A). Kaplan–Meier survival analysis further demonstrated that patients with higher DNMT1 expression levels in glioma exhibited lower overall survival rates, disease-specific survival rates, and shorter progression-free periods compared to patients with lower DNMT1 expression levels (Fig. 7B). Clinical correlation analysis indicated a positive correlation between DNMT1 expression and the WHO grade of glioma, with the highest expression observed in grade 4 gliomas and the lowest expression in grade 2 gliomas. Additionally, the expression of DNMT1 was associated with clinical characteristics, including patient age, histopathological type, and prognosis following initial treatment (Fig. 7C).

In the present study, bioinformatics analysis was performed using DNMT1 co-expressed genes in glioma. The top 300 co-expressed genes associated with DNMT1 were identified from the TCGA database. GO analysis revealed that DNMT1 was involved in various BP, including extracellular matrix composition, peptidase regulatory activity, peptidase inhibitor activity, growth factor binding, organelle fission, nuclear division, and centriole replication. KEGG pathway analysis indicated that DNMT1 was involved in signaling pathways, such as systemic lupus erythematosus, the cell cycle, ECM–receptor interaction, and the p53 pathway (Fig. 7D, E). These findings suggested that DNMT1 might play a critical role in the progression and prognosis of gliomas.

### NUP37 expression in glioma is positively correlated with and interacts with DNMT1

**Fig. 8:**
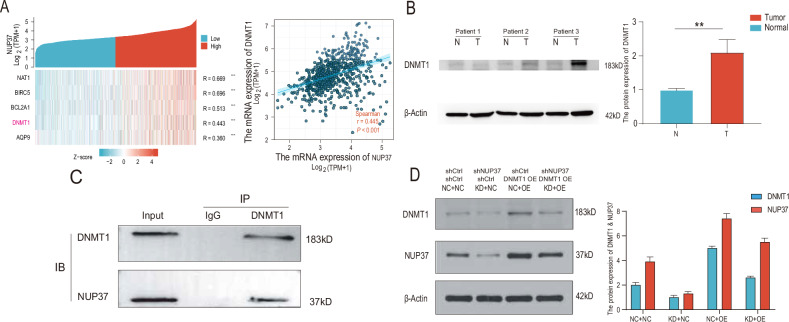
DNMT1 is overexpressed in glioma cells, NUP37 promotes DNMT1 expression in glioma, and there is an interaction between DNMT1 and NUP37. **A** The correlation between NUP37 and DNMT1 in gliomas was analyzed based on the TCGA public database. **B** The expression level of DNMT1 in glioma tissues was detected by western blotting analysis. **C** Western blotting analysis was used to detect the expression of DNMT1 after NUP3 depletion. **D** Co-IP was used to verify the interaction between NUP37 and DNMT1. The significance level was set as follows: **P < 0.01.

Correlational analysis of glioma data from the TCGA database demonstrated a positive association between the expression levels of NUP37 and DNMT1 (Fig. 8A). The western blotting analysis indicated that DNMT1 was substantially overexpressed in glioma tissue relative to normal brain tissue (Fig. 8B). Furthermore, findings from the Co-IP study suggested a potential interaction between NUP37 and DNMT1 (Fig. 8C).

### DNMT1 overexpression restores some of the declines in the biological function of glioma cells caused by NUP37 depletion

In our prior cellular function experiments, we found that NUP37 depletion in glioma cells significantly curtailed cell proliferation and invasion, triggered apoptosis, induced nuclear migration, and led to various degrees of cell cycle arrest. To delve deeper into the role of DNMT1 in these cellular functions, we overexpressed DNMT1 in NUP37-depleted glioma cells and assessed the changes in cell functions (Fig. 8D).

**Fig. 9:**
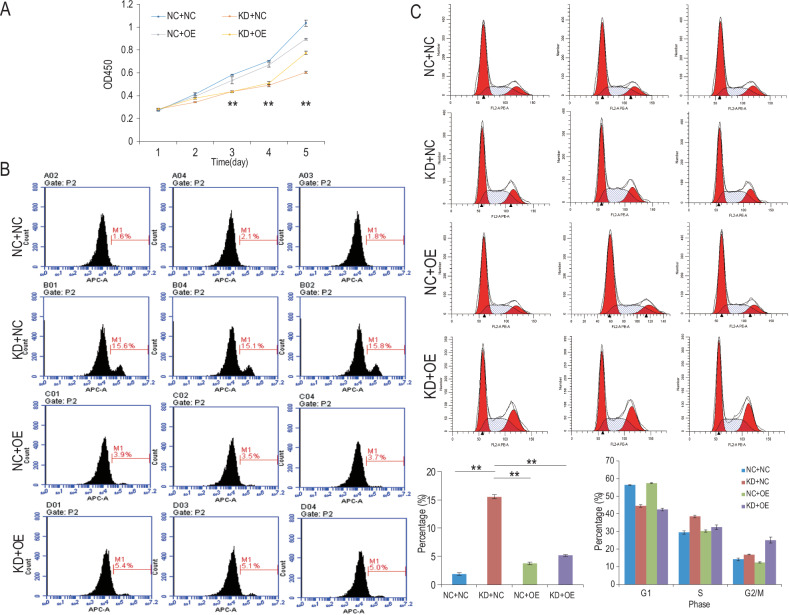
Overexpression of DNMT1 can rescue the adverse effects of NUP37 depletion on the biological functions of glioma cells. **A** CCK-8 assay was used to detect the proliferation activity of glioma cells after DNMT1 overexpression. **B** Flow cytometry was used to detect the apoptosis rate of glioma cells after DNMT1 overexpression. **C** Flow cytometry was used to detect the proportion of U87 and U251 glioma cells in each cycle after DNMT1 overexpression. The significance level was set as follows: **P < 0.01.

**Fig. 10:**
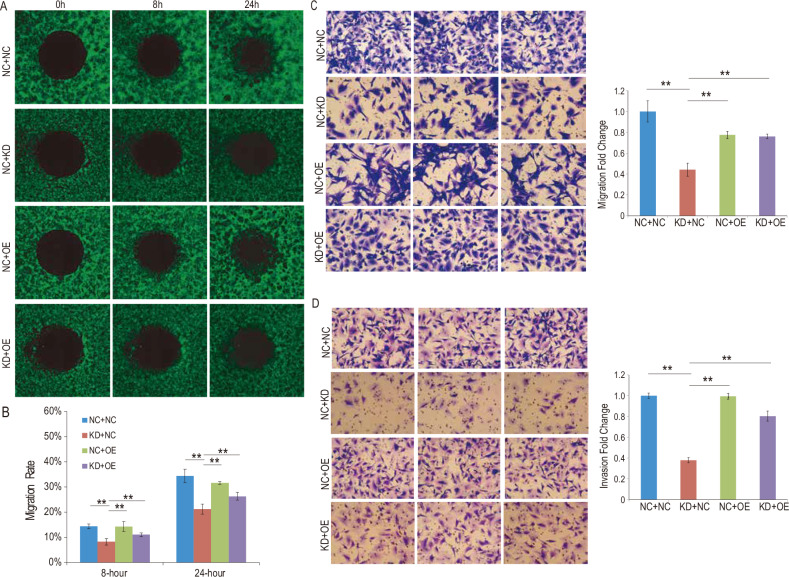
Overexpression of DNMT1 can rescue the adverse effects of NUP37 depletion on the biological functions of glioma cells. **A**, **B** Oris™ Wound healing assay was used to detect the migration ability of glioma cells after DNMT1 overexpression. **C**, **D** Transwell migration and invasion assay was used to observe the invasion and migration level of glioma cells after DNMT1 overexpression. The significance level was set as follows: **P < 0.01.

The CCK-8 cell proliferation assay revealed that the proliferation activity of the glioma cells reached its peak in the double-negative control group, while it was at its lowest in the group when NUP37 was depleted. Furthermore, the proliferation capability of glioma cells was higher in both DNMT1-overexpressing groups compared to the NUP37-depleted group (Fig. 9A, B). Similarly, the Oris™ Wound Healing Assay findings revealed that the migration level of glioma cells was greatest in the double-negative control group, least in the NUP37-depleted group, and higher in both DNMT1-overexpressing groups compared to the NUP37-depleted group (Fig. 10A).

In the apoptosis experiment, the apoptosis ratio of the glioma cells was found to be the lowest in the double-negative control group and the highest in the NUP37-depleted group. Additionally, the apoptosis ratio of glioma cells in both DNMT1-overexpressing groups was observed to be higher than that in the NUP37-depleted group (Fig. 10B). Correspondingly, the results of the Transwell invasion and migration experiments mirrored these conclusions. The levels of invasion and migration of glioma cells peaked in the double-negative control group, reached their lowest in the NUP37-depleted group, and were elevated in both DNMT1-overexpressing groups when compared to the NUP37-depleted group (Fig. 10C, D). Moreover, variations in the cell cycle of glioma cells were observed across the four experimental groups (Fig. 9C). To summarize, in glioma cells with NUP37 depletion (NC + KD), overexpression of DNMT1 (KD + OE) partially restored the proliferation, invasion, and migration of glioma, while it decreased apoptosis to varying degrees.

### The upregulation of DNMT1 causes the methylation of genes related to cell proliferation and signal transduction regulation

**Fig. 11:**
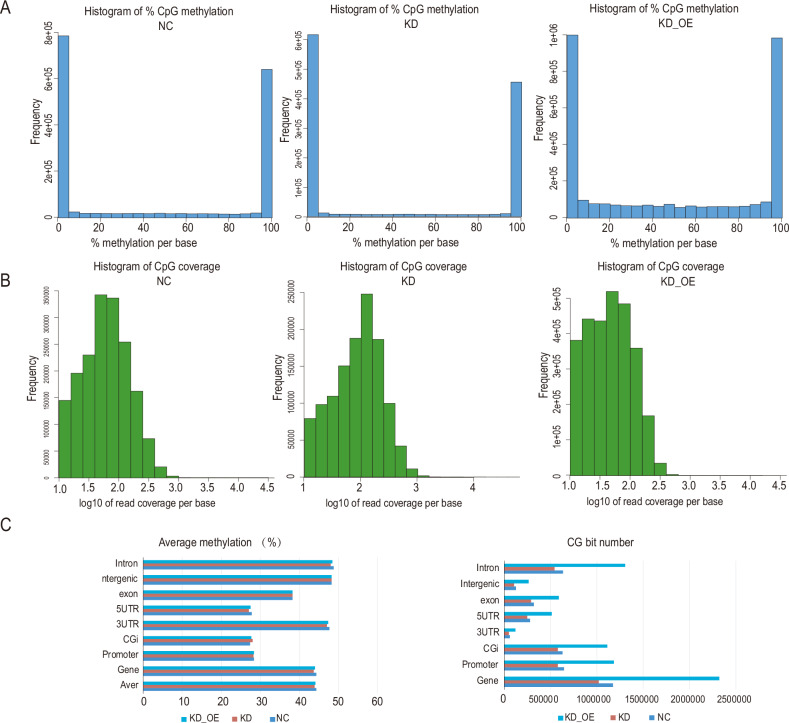
The increased expression of DNMT1 under NUP37 knockdown led to the increased methylation of several cell signaling pathway genes related to cell proliferation and invasion. **A** CpG distribution at different methylation levels in glioma cells after NUP37 knockout. **B** CpG site coverage in glioma cells after NUP37 knockout. **C** Distribution of CpG and methylation distribution of CpG in functional elements.

The findings from reduced representation bisulfite sequencing methylation sequencing showed a decrease in the methylation rate of CpG sites in glioma cells following NUP37 depletion (Fig. 11A, B). This observation aligned with the results obtained from transcriptome sequencing. Conversely, overexpression of DNMT1 led to a significant increase in the methylation rate of CpG sites within glioma cells. Additionally, the enrichment analysis of DEGs indicated changes in the expression of pathways related to cell proliferation and cell–cell signal transduction following DNMT1 overexpression (Fig. 11C). This evidence suggested that DNMT1 might engage in cell proliferation-related pathways to preserve the biological function of certain glioma cells under the condition of NUP37 depletion.

## Discussion

Glioma constitutes a significant hazard to human health, with its 5-year survival rate only surpassed by pancreatic and lung cancers, thereby exerting a substantial societal and familial burden [20, 21]. In recent years, a steady increase in glioma incidence has been noted, along with an alarming trend of younger individuals being affected. The pathogenesis of glioma is multifaceted, encompassing alterations in gene expression and protein modification. However, the underlying mechanisms of glioma initiation and progression remain largely elusive, potentially implicating environmental factors and genetic mutations. At present, well-established risk factors for glioma primarily include exposure to high-dose ionizing radiation and high-penetrance genetic mutations linked to rare syndromes [22].

Alterations in cell cycle regulation underpin the unchecked growth of tumor cells [13]. A hallmark of tumor progression is the dysregulation of cell cycle progression or apoptosis in normal epithelial cells, leading to unrestrained proliferation [23], a crucial factor in tumor recurrence [24]. Inhibiting the cell cycle can effectively constrain the proliferation and division of tumor cells to varying extents [25], thereby delaying tumor recurrence. Previous studies have indicated that NUP37 fosters tumorigenesis and development by modulating the cell cycle of lung cancer cells [22].

In the present study, we performed cell cycle assays to examine the impact of NUP37 depletion on the cell cycle of U87 and U251 glioma cell lines. The findings revealed that NUP37 depletion induced cell cycle arrest to varying degrees. Specifically, NUP37 depletion caused arrest at the G2/M phase in U87 cells, while in the U251 cell line, varying degrees of G1 phase arrest were observed. These results suggested that NUP37 overexpression might exert regulatory influence on the cell cycle of glioma cell lines, potentially fueling the rampant proliferation of glioma cells and aiding in tumor formation.

Another pivotal phase in tumor metastasis is the migration and invasion of tumor cells [26, 27], and the highly aggressive nature of glioma presents a significant challenge in its treatment [28]. Effectively curtailing tumor cell invasion can decelerate tumor progression and prolong patient survival [29, 30]. Previous studies have illustrated that the inhibition of NUP37 can significantly reduce the invasion and migration of liver cancer cells using biological inhibitors [31]. In this study, we further explored the impact of NUP37 depletion on the invasion and migration of glioma cells. Our findings indicated that the capabilities of invasion and migration were markedly influenced by NUP37 depletion in U87 and U251 glioma cell lines. These results corroborated the findings from the preceding bioinformatics analysis, suggesting that NUP37 might contribute to glioma invasion and metastasis.

Methylation imbalance is a hallmark of most malignant tumors, including glioma. A wealth of research has underscored the crucial role of aberrant methylation regulation in the onset and prognosis of glioma. Deviant methylation patterns have been linked with tumorigenesis, with differential methylation profiles reported between tumor and benign tissues across various types of cancer [32, 33]. DNA methylation in the promoter region can induce gene silencing, leading to the inactivation of tumor suppressor genes, an epigenetic alteration of significant relevance in glioma [34, 35].

As an inheritable and modifiable epigenetic adjustment, methylation’s underlying mechanisms have been explored as potential targets for glioma treatment in recent years [36, 37]. DNA methylation inhibitors have found their use in the clinical treatment of specific tumors. For instance, decitabine serves as a primary chemotherapy drug for hematological tumors associated with myelodysplastic syndrome [38–40].

In the field of glioma treatment, strategies that target DNA methylation have also been explored. Temozolomide, a commonly employed chemotherapy drug, is recommended as a frontline treatment in official guidelines for the diagnosis and management of gliomas. It induces DNA methylation in tumor cells, leading to the formation of O6- and N7-methylguanine lesions. These lesions hinder DNA synthesis and repair, thereby inhibiting tumor growth [41]. Methylation of the O6-methylguanine-DNA methyltransferase promoter can also decrease DNA repair capacity, rendering tumor cells more susceptible to the effects of temozolomide.

In conclusion, our study underscored the significance of NUP37 and DNMT1 in the regulation of glioma proliferation. We noted overexpression of NUP37 and DNMT1 in glioma tissues. Through extensive bioinformatics analysis and multi-omics sequencing, we ascertained that NUP37 overexpression in glioma led to an increase in various cell proliferation, division, and cell cycle-related signaling pathways. Moreover, we illustrated that DNMT1 overexpression, in the context of NUP37 depletion, could partially restore both the proliferation and invasion of glioma cells. Integrating these findings with previous sequencing data and cellular functional assays, we proposed that NUP37 depletion induced DNMT1 downregulation, thus inhibiting glioma cell proliferation and invasion.

Based on our findings, targeting DNMT1-related demethylation pathways could offer a potential therapeutic strategy for glioma treatment. Furthermore, continued investigations into the interaction between NUP37 and DNMT1, as well as their specific signaling pathways and molecular mechanisms, will enhance our understanding of glioma pathogenesis and broaden the therapeutic arsenal for this devastating disease.

## Conclusion

These findings suggested that high expression of NUP37 regulated the proliferation and invasion of glioma cells by binding DNMT1.

## Materials and methods

### Cells and human tissue samples

The U87 and U251 cell lines were purchased from Shanghai Genechem Co., Ltd. Both U87 and U251 cells were maintained in DMEM supplemented with 10% bovine serum, penicillin (100 U/mL), and streptomycin (100 U/mL) at 37 °C in a humidified atmosphere containing 5% CO_2_.

We collected 35 cases of human gliomas from the neurosurgical specimens at the First People’s Hospital of Changzhou. All patients underwent surgery for the first time, and all cases were pathologically confirmed as glioma. Additionally, normal brain tissues were obtained from 15 patients who experienced traumatic brain injuries. The study received approval from the Research Ethics Committee of the First People’s Hospital of Changzhou. All patients involved in the study provided informed consent prior to their participation, and all specimens were subjected to anonymous processing in accordance with ethical and legal standards.

### Construction of NUP37 knockdown and DNMT1 overexpression lentivirus

Stable gene depletion in U251 and U87 cell lines was achieved by infecting the cells with recombinant small hairpin RNA (shRNA)-expressing lentiviruses. The lentiviral vectors containing shRNA targeting NUP37 and negative control (NC) were obtained from Genechem (Shanghai, China), followed by selection with puromycin at a concentration of 5 mg/mL for ~2 weeks. The target sequences for the shRNA are as follows: NUP37, 5′-TTGCCTCCAGTAATCAAAT; DNMT1, 5′-GCATCCTTCAATTTCTGTATA. The selected infected glioma cells were subsequently used for various experiments, including cell functional tests, mouse model experiments, transcriptome sequencing, proteomics sequencing, and recovery experiments.

### Quantitative RT-PCR

Total RNA was extracted from tissue specimens and cells using a Trizol kit. Subsequently, the RNA concentration and purity were determined to prepare the necessary template for PCR. A standard quantitative RT-PCR was carried out as previously described, and the relative expressions of the target genes were calculated using the 2^−ΔΔCT^ method. The primers used were as follows: for NUP37, forward: 5′-TTTTAGAGGGCCATACCGATTTC, and reverse: 5′-AAACCGGATTGTTCCATTCTTCT; for ACTB, forward: 5′-GCGTGACATTAAGGAGAAGC, and reverse: 5′-CCACGTCACACTTCATGATGG.

### Western blotting analysis

Glioma and peritumoral tissues, as well as total cell lysates, were lysed in RIPA lysis buffer (Beyotime, China) supplemented with a complete protease inhibitor cocktail (Roche, USA). Protein concentration was determined using a Protein Assay Kit (Beyotime, China). Briefly, equal amounts of proteins (20 µg) were subjected to sodium dodecyl sulfate-polyacrylamide gel electrophoresis (SDS–PAGE) and then transferred onto a polyvinylidene difluoride membrane (Roche, USA). The membrane was incubated with TBST containing 5% skim milk for 2 h at room temperature. Subsequently, the blot was incubated with primary antibodies, including rabbit anti-Nup37 (1:10,000, Abcam, ab185230), rabbit anti-DNMT1 (1:10,000, Abcam, ab185230), and rabbit anti-beta-actin (1:2000, Abcam, China), overnight at 4 °C. The membrane was then incubated with a goat anti-rabbit monoclonal IgG secondary antibody (1:100,000; Abcam, China) for 2 h at room temperature. The immunoreactive bands were visualized by chemiluminescence using an ECL kit (Genshare Biological, China).

### Co-IP assay

Cells were prepared, and proteins were subsequently extracted using RIPA reagent. The extracted proteins were equally divided into two 1.5-mL Eppendorf tubes. The first tube was treated with the target antibody (anti-DNMT1), while the second one was treated with a corresponding rabbit antibody from the same species. Both tubes were then incubated at 4 °C overnight on a gentle rocker. Subsequently, ProteinA/G magnetic beads were added to both tubes to capture the immunoprecipitated complex. The targeted proteins were then detected using western blotting analysis.

### Cell proliferation assay and Celigo plate count analysis

In this study, the impact of NUP37 silencing on cell proliferation was assessed using the MTT Assay Kit (Sigma) as previously described [16]. In brief, U87 and U251 cells, after lentiviral infection, were seeded into 96-well plates at a density of 3000 cells/well. On days 1, 2, 3, 4, and 5, 10 μL of MTT solution was added to each well, followed by a 4-h incubation at 37 °C. The absorbance was then measured at 570 nm using an automated microplate reader (BioTek, Winooski). After digestion with trypsin, cells from the experimental groups in the logarithmic growth phase were resuspended in a complete medium and enumerated under a microscope. These cells were then seeded into 96-well plates at a density of 2000 cells/well, each well containing 100 μL of the medium. Each group was replicated in triplicate, distributed across five 96-well plates, and cultured in an incubator. Ten microliters of CCK-8 reagent was added to each well 2–4 h before the day’s incubation period ended. The optical density (OD) was then measured using a microplate reader at 450 nm.

U87 and U215 cells transfected with shNUP37 or control shRNA were detached using trypsin, resuspended, and then seeded into 96-well plates. For 5 consecutive days, fluorescence emission was captured using the Celigo^®^ system. Specific alterations were implemented in the analysis settings to convert fluorescence expression into corresponding cell counts.

### Flow cytometry

To evaluate the effect of NUP37 on cell apoptosis, cells transfected with either shNUP37 or control shRNA were harvested and washed with PBS. Apoptosis was then assessed using the Annexin-V/FITC Apoptosis Detection Kit (Invitrogen, USA) as per the manufacturer’s instructions. For cell cycle analysis, cells were fixed in 70% ice-cold ethanol at −20 °C overnight. Following fixation, cells were incubated with PBS containing 50 μg/mL of propidium iodide (PI, Sigma) at 37 °C for 15 min. The samples were subsequently analyzed using flow cytometry. This allowed us to investigate whether the silencing of NUP37 resulted in changes to the cell cycle distribution, which could, in turn, impact cell proliferation.

### Cell invasion and migration assay

The invasion capacity was measured using a Matrigel-coated Transwell invasion chamber. Cells from different groups (1 × 10^6^ cells/well) were collected and cultured in the upper chamber without serum for 24 h, while the medium containing 10% FBS served as a chemotactic agent in the lower chamber. After 24 h of culture, the medium in the upper chamber was discarded, and cells that migrated to the lower chamber were collected and resuspended. Any cells that did not migrate were removed with cotton swabs, and then the migrated cells were fixed with formaldehyde and stained with crystal violet. Under a DM-2500 microscope (LEICA, Germany), five visual fields were randomly selected to calculate the number of migrating cells. The Transwell migration assay procedure was essentially identical, except for the lack of Matrigel matrix in the invasion chamber, making it less invasive.

### In vivo tumor-bearing assay

All animal experiments were approved by the Ethics Committee of the First People’s Hospital of Changzhou. BALB/c nude mice, procured from Shanghai Lingchang Biotechnology Co., Ltd. (Shanghai, China), were housed under standard conditions. Ectopic tumor mouse models were established by inoculating U87 glioma cells from both the experimental group (shNUP37) and the control group (shCtrl) into the subscapular area of the mice. The tumor volume was measured at least twice per week. On the 21st day, the mice were anesthetized and subsequently sacrificed. The tumors were then excised and weighed, and the volumes were recorded for statistical analysis.

### RNA sequencing and proteomics sequencing

Lentiviral transfection was employed to suppress NUP37 in glioma cells. Total RNA was extracted from the treated glioma cells using the Trizol method, followed by the detection of its concentration and purity. The NEBNext RNA kit was utilized for library construction. Upon completion, quantitative and qualitative inspections were performed using appropriate software. Transcriptome sequencing was conducted on the Illumina platform, and the resultant data were organized and analyzed. In a similar vein, lentiviral transfection was used to deplete NUP37 in glioma cells. The RIPA method was employed to extract the total protein from glioma cells post-NUP37 treatment, and the BCA method was utilized to determine protein concentration. The extracted protein samples were hydrolyzed into peptides by FASP enzymatic hydrolysis and peptide quantification. Following chromatographic separation, the samples were detected and analyzed by mass spectrometry using the PASEF mode on a timsTOF Pro mass spectrometer. The data were then sorted and analyzed. Both transcriptome sequencing and proteomics sequencing were carried out by Shanghai Genechem Co., Ltd.

### Public data source acquisition and analysis

TCGA (https://genome-cancer.ucsc.edu/), a complimentary large-scale cancer genome project data portal, offers clinical and pathological information on 33 types of cancer for academics and researchers. Genotype-Tissue Expression (GTEx), like TCGA, is also free to the public. However, TCGA focuses more on tumor-related data, whereas GTEx collates tissue samples from healthy individuals for sequencing. NUP37 expression profiles, along with TCGA and GTEx clinical pan-cancer data, were downloaded from the University of California, Santa Cruz (UCSC) Xena database (https://xenabrowser.net/datapages/), then processed and analyzed to evaluate NUP37 expression. Pearson correlation analysis of NUP37 mRNA and other mRNAs was conducted in glioma using TCGA GBM data. The 300 genes most positively associated with NUP37 were chosen for enrichment analysis to determine NUP37’s function. Following this, GO and KEGG pathway analyses were performed using the clusterProfiler and ggplot2 packages in R software. Furthermore, DRSEQ2 and Clusterprofiler in R software were used to carry out single-gene differential analysis and GSEA enrichment analysis on TCGA GBM data, respectively.

### Statistical analysis

Statistical data obtained from TCGA were consolidated and processed using R-3.6.3. The correlations between clinical information and NUP37 expression were examined through logistic regression. Furthermore, multivariate Cox analysis was employed to evaluate the impact of NUP37 expression and other clinicopathological factors, such as age and gender, on survival rates. A *P* value of <0.05 was established as the cut-off criterion. All experiments were conducted in triplicate. The results are represented as mean ± the standard deviation (SD). Statistical significance was determined using a one-way analysis of variance (ANOVA) test. The levels of significance were designated as follows: *P < 0.05, **P < 0.01, and ***P < 0.001.

### Supplementary information

Full length western blots

## Data Availability

All data generated or analyzed during this study are included in this published article.
