# Supplementary figures and images for: NUP37 promotes the proliferation and invasion of glioma cells through DNMT1-mediated methylation
Source: Cell Death Discov. 2024 Aug 22;10:373. doi: 10.1038/s41420-024-02138-5 (PMC11341718; doi:10.1038/s41420-024-02138-5)

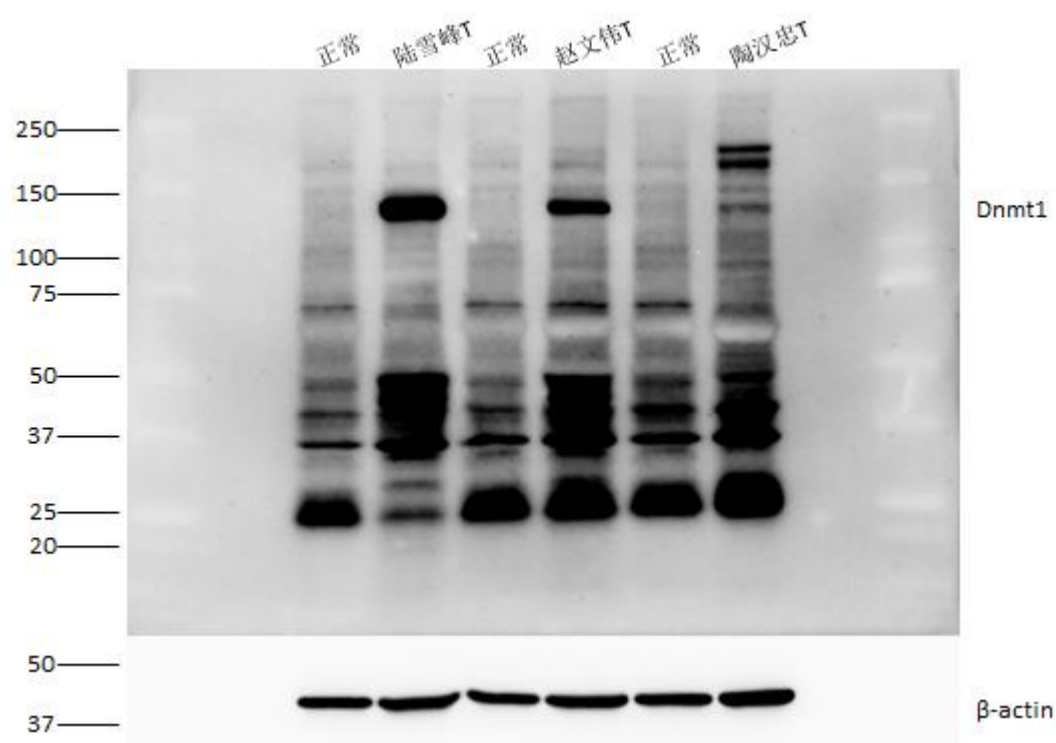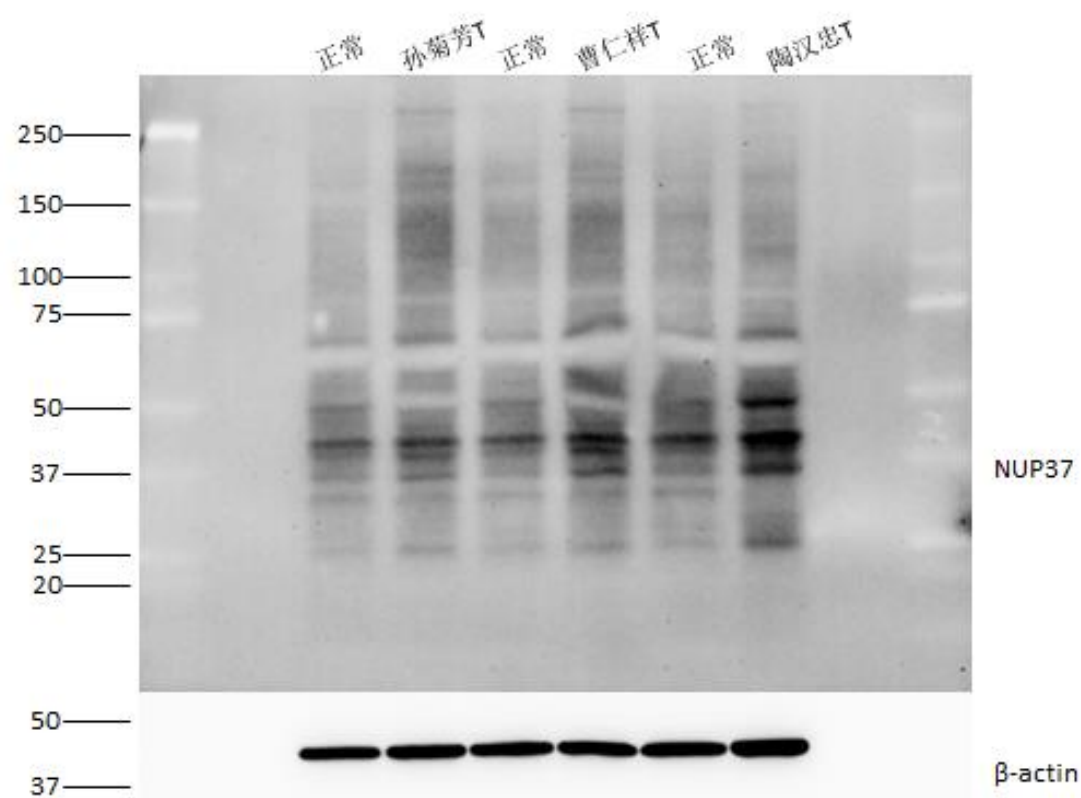

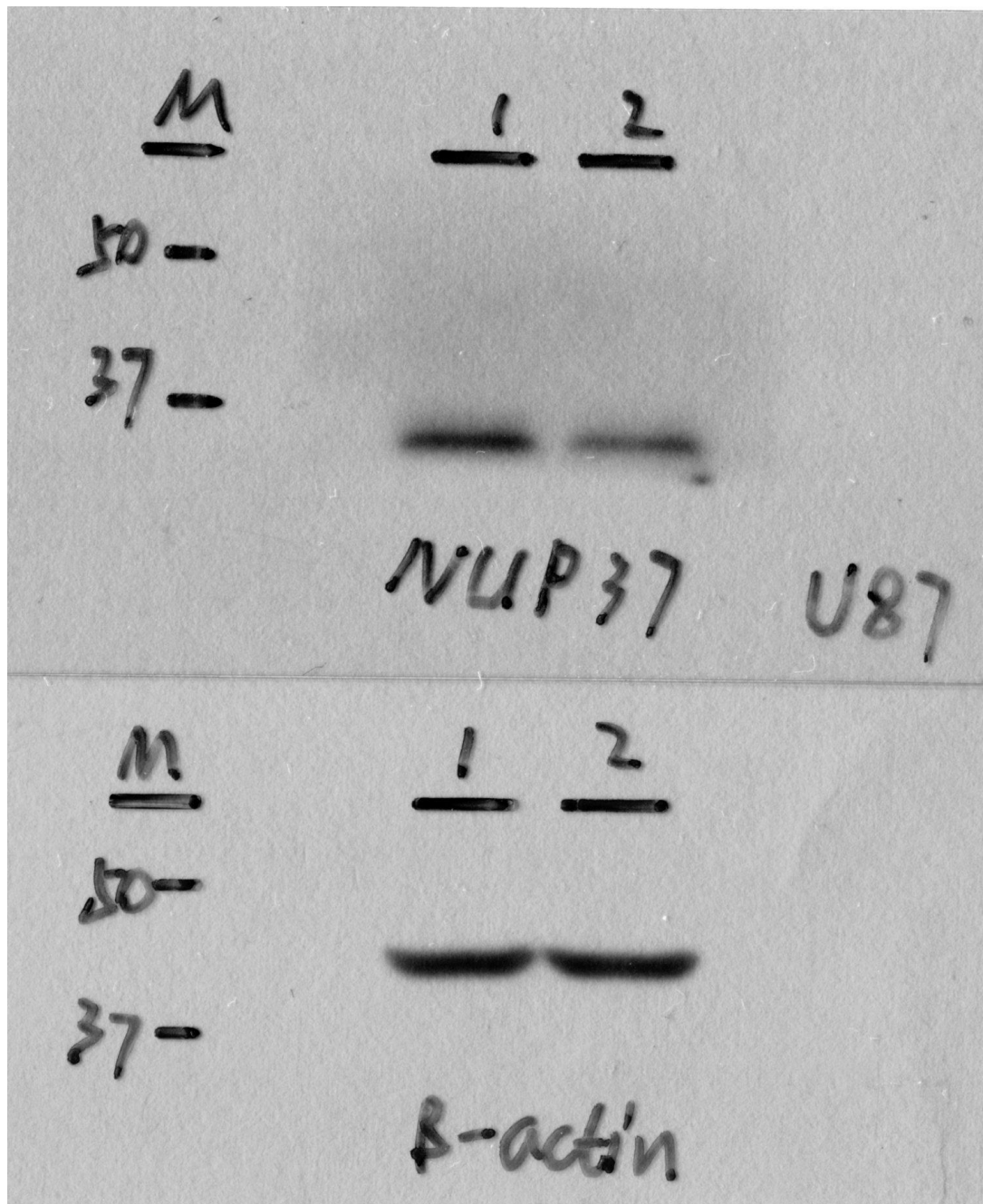

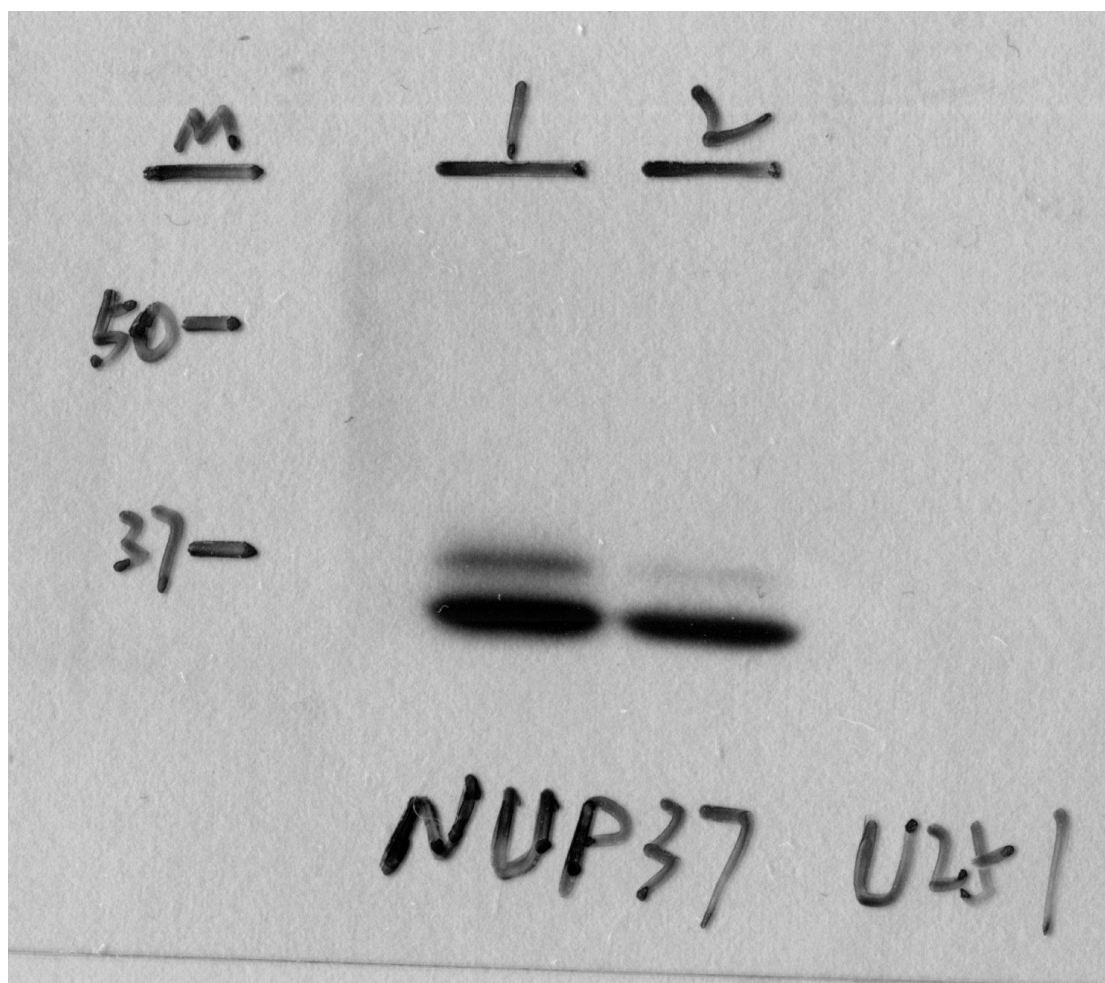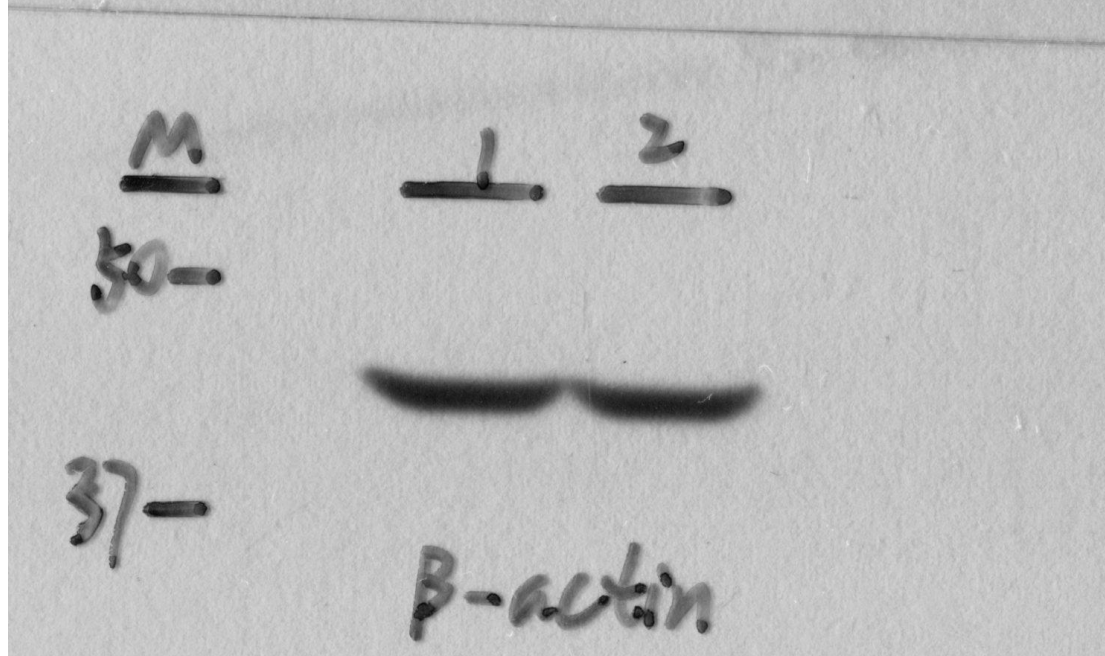

Supplement: Supplementary file 1 — Full length western blots [file 41420_2024_2138_MOESM1_ESM.pdf]
